# The Emergence of Groups and Inequality through Co-Adaptation

**DOI:** 10.1371/journal.pone.0158144

**Published:** 2016-06-30

**Authors:** Jon Atwell, Robert Savit

**Affiliations:** 1 Department of Sociology, University of Michigan, Ann Arbor, MI, United States of America; 2 Center for the Study of Complex Systems, University of Michigan, Ann Arbor, MI, United States of America; 3 Department of Physics, University of Michigan, Ann Arbor, MI, United States of America; Georgia Institute of Technology, UNITED STATES

## Abstract

The emergence of groups and of inequality is often traced to pre-existing differences, exclusionary practices, or resource accumulation processes, but can the emergence of groups and their differential success simply be a feature of the behaviors of *a priori* equally-capable actors who have mutually adapted? Using a simple model of behavioral co-adaptation among agents whose individual actions construct a common environment, we present evidence that the formation of unequal groups is endemic to co-adaptive processes that endogenously alter the environment; agents tend to separate into two groups, one whose members stop adapting earliest (the in-group), and another comprising agents who continue to adapt (the out-group). Over a wide range of model parameters, members of the in-group are rewarded more on average than members of the out-group. The primary reason is that the in-group is able to have a more profound influence on the environment and mold it to the benefit of its members. This molding capacity proves more beneficial than the persistence of adaptivity, yet, crucially, which agents are able to form a coalition to successfully exert this control is strongly contingent on random aspects of the set of agent behaviors. In this paper, we present the model, relevant definitions, and results. We then discuss its implications for the study of complex adaptive systems generally.

## Introduction

Where it exists, inequality between groups is often traced to pre-existing differences [1], exclusionary practices [2][3], or resource accumulation processes [4][5][6][7][8], but can the emergence of groups and their differential success simply be a feature of the behaviors of *a priori* equally-capable actors who have mutually adapted? Using a simple model of behavioral co-adaptation among agents whose individual actions construct a common environment [9], we present evidence that the formation of unequal groups is endemic to co-adaptive processes that endogenously alter the environment: agents tend to separate into two groups, one whose members stop adapting earliest, and another comprising agents who continue to adapt.

The evidence of this dynamic is intriguing because in a variety of settings, actors change their behavior with the goal of being better adapted to their environment, but at the same time their adaptive behaviors alter that environment. A collection of actors occupying the same environment is thus thrown into a complex dance of co-adaptation via the environment they construct. Humans and many other species have the ability to modify their local environment in ways that alter, intentionally or not, their prospects for success in that environment, a process often referred to in evolutionary theory as niche construction [10][11]. (See [12] for a debate on the history of this concept.) While this process can and does happen on an evolutionary time scale, it can be fruitfully studied without reference to genetic evolution. Co-adaptation can happen on a shorter time scale because individual efforts to exhibit successful behavior will often result in changes to the shared environment that force others to quickly adapt. In biology, for example, niche-constructing behavior ranges widely, from the engineering activities of soil fauna [13] to intricate behaviors such as the trip wires constructed by Corolla spiders [14][10]. A similar dynamic is present in the network of mostly autonomous internet routers tasked with getting data from a source to its destination efficiently. The choice of path is a function of the throughput on the possible routes, which is determined by all routers' previous choice of paths [15][16]. The overall flow of traffic emerges from the co-adaptive behavior of individual routers to their mutual constructed traffic environment [17][18].

Humans and other species of high cognitive abilities often adapt to a physical and social environment of their own making. While physical environments can be affected [19][20][21], behavioral co-adaptation is often more clearly present in social environments; individuals are able to construct shared meanings and evaluations of actions, words, and objects by adapting their understanding and use of them to their use by others [22][23][24]. This co-adaptation of individual interpretive actions and the social environment is key to the emergence of shared understandings and other elements of culture [25][26][27][28][29]. Consumer behaviors can exhibit a similar dynamic as essential information about goods and services can be transmitted indirectly via a shared environment.

Much of the formal research on co-adaptation and coordination is focused on direct interaction and understates the role of endogenously structured environments, and this omission, when unjustified, can result in misleading conclusions. An endogenously structured environment will have different general characteristics than an exogenously structured one and will raise new and different questions. For example, when one cannot assume a fixed set of costs and benefits for individuals`actions relative to the environment (e.g. the values in a payoff matrix), one ought to ask if there are systematic changes to those costs and benefits [30][31][32] and whether distinct relationships among agents form in the face of such changes. One might also study the capacity of the environment to carry information about the behavior of others and whether this alters direct agent-to-agent interactions. Questions about equilibria and systemic robustness in the face of exogenous shocks are also likely to yield different answers because endogenous influences on the environment will lead to a different dynamics of agent-mediated environmental response. In this paper we present evidence that endogenous molding tends to result in functionally distinct groups with unequal success in the mutually constructed environment.

In order to better understand the process of endogenous molding of the environment, we focus on the abstract mechanism in isolation. Of course, real world settings such as those we have mentioned above (genetic evolution in the case of niche construction, human design and intervention in the case of internet traffic routing, or social networks, group-affiliations, and interpersonal learning in the case of human society) may exhibit additional sources of, or constraints on, the structure of the environment. While any empirical work must to take into account the range of mechanisms present, those efforts will be well-served by the explicit and detailed understanding of the features of a simplified version of endogenous co-adaptation such as that embodied in the model we study here, first proposed by Savit, Riolo and Riolo (SRR)[9] and referred to as the stigmergy game.

In this model, the only possible mechanism for communication and, consequently, for co-adaptation among the agents is *stigmergy*, that is, signaling through the (endogenously constructed) environment. The naturalist Pierre-Paul Grasse coined the term stigmergy to describe settings in which social insects “interact indirectly when one of them modifies the environment and the other responds to the new environment at a later time”(pp. 14) [33]. Recent research has shown the mechanism of stigmergy to be quite common [34][35] and robust [28][9], suggesting it plays an important role in the emergence of tacit agreements about interdependent behavior. The SRR model was designed to study the meta-dynamics of a wholly endogenously structured environment without any direct agent-to-agent communication and asks whether that process can transmit enough information for coordination to emerge among the agents. Emergent coordination here is understood as better than zero-sum mutualisms among the set of agents, so that on average agents benefit more than they would have without co-adaptation. SRR showed such mutualisms are possible and common. This fact raises the important question of whether the emergent mutualisms involve and benefit agents equally, or if instead the dynamics of such co-adaptation produces differentiation among agents. The tremendous diversity of behaviors present in the natural and social worlds suggests the latter and therefore we sought to understand the ways in which the agents in the stigmergy game might become functionally differentiated.

In this paper, we show that a consistent dynamic underlying the coordination identified in SRR is the emergence of a subset of agents that is able to direct the structure of the shared environment toward having characteristics desirable to it, while the individuals in the complementary group struggle to adapt and extract fewer rewards on average than those in the first group. We call these groups the *in-group* and the *out-group*, respectively. Although there are some counter-examples, this underlying dynamic of differential success between the groups is robust. As we discuss below, this robust dynamic has a number of important implications for endogenous co-adaptation and for complex adaptive systems in general. The rest of this paper is organized as follows: In the next section we will briefly summarize the stigmergy game and its most general behaviors. [Readers familiar with the stigmergy game can skip this section. For a more complete discussion, see SRR.] We then present a formal definition for the groups and show that members of the in-group generally acquire more rewards than members of the out-group. The paper ends with a discussion of the results and broader implications.

## The Stigmergy Game

### Model Specification

The stigmergy game models *N* agents adapting to each other indirectly by taking turns responding to one of *E* possible states for a shared environment. Every turn starts with an agent being randomly selected to observe the current state of the environment (say, e_1_) and transform it to a new state (say, e_2_). (Random selection permits an agent to be selected multiple times in a row or wait long periods without acting.) The acting agent receives a reward of +1, -1 or 0 based on the state of the environment they first observed. The state transformation and reward are determined by a “strategy,” a look-up table of *E* rows and 3 columns. Table 1 is an example for *E* = 5. The first column lists all *E* possible input states of the environment, the second determines the new environmental state given an input state, and the third determines the reward. The second column contains randomly (identically and independently) distributed instances of all possible environmental states. The third is also randomly distributed but with the constraint that it sums to zero so that each strategy is reward neutral, thus controlling for an obvious source of advantage. Using the strategy it is currently playing, an agent reads across the row associated with the environmental state it sees on its turn to determine the appropriate transformation and reward. For example, an agent using the strategy in Table 1 who observes state 5 would transform the environment to state 3 and gain a payoff of +1.

**Table 1:** An example strategy for E = 5.

Current State	New State	Reward
1	5	-1
2	2	+1
3	4	-1
4	2	0
5	3	+1

An agent is randomly assigned *S* strategies from among all possible strategies. Because the number of possible strategies is large for even moderate values of E, different agents have different sets of strategies. After each of its turns, an agent selects the strategy in its set that, had it been used for every one of the agent’s previous turns, would have the largest cumulative rewards. (An agent updates a hypothetical cumulative reward [score] for all its strategies every time it acts. Older rewards are not discounted because our analysis of “memory” discounting revealed no effect except for very short memories. This is likely because the strategies’ scores quickly become separated by nontrivial amounts as the environment becomes more orderly). Ties between the agent’s strategies are broken randomly. Strategies are reward neutral in a uniform distribution of environmental states and randomly assigned in order to best isolate the emergent features attributable to the dynamics of co-adaptation.

In this model, an agent molds the environment by changing its state according to the rules described in the agent’s current strategy. If an agent ceases to change strategies it is nonetheless actively producing the states of the environment that agents see and therefore has an ongoing role in the molding the shared environment. In fact, because it has stopped switching strategies, it is more consistently molding the environment since, for example it is invariably producing state *e*_*2*_ when it sees state *e*_*1*_. Furthermore, that an agent might settle on a particular strategy means that the distribution of environmental states the agent sees is better matched to that strategy than any other it has and, therefore, that the environment has been successfully *molded* by the collection of all agents to that agent and its behavior. This dynamic of self-reinforcing adaptation is at the core of the model and is what we mean by endogenous molding. The process is sufficiently complex that it is not obvious how to formally characterize the dynamics and equilibria as one might do in game theory. The challenges to molding the environment are enough that it is not clear a group of agents can even coordinate to produce a mutually beneficial environment. SSR showed that it is possible, however, and we now review the metrics and findings that relate to our present inquiry.

### Summary of Metrics and General Behavior

The general behavior of this model is described in detail in SRR. Here we excerpt the description of three metrics—average reward, environmental order, and switching rate—and present a notional figure indicating the general qualitative behavior of the system as a function of *N* and *E*.

#### Average agent wealth

We track agents’ “wealth” (i.e. sum of rewards) over a time window to find a per turn average reward. The average agent wealth reported here is the group average of this individual reward rate over the same window. This rate is between [1, –1] with an expected value of zero when states are equally probable. Specifically, the rate of wealth accumulation by agent *i* over a window of *T* time steps can be written as

$$W\left(i\right)=\frac{1}{\tilde{T}}\sum_{\tau =t_{0}}^{t_{0}+T}\prime \;r_{j\left(\tau \right)}\left(i;e(\tau )\right),$$ (1)

The times agent *i* acts are denoted by *τ* and the prime on the sum indicates that it includes only those time steps. $\tilde{T}$ is the total number of times agent *i* acts in the interval [t_0_, t_0+T_]. When the *i*^*th*^ agent uses its *j*^*th*^ strategy at time τ, r_j(τ)_(i; e(τ)) is the entry in the reward column of the *j*^*th*^ strategy associated with the input state the agent sees at time τ, e(τ). W(i) can be understood as a measure of an agent`s success adapting to the behavior of others. An average over the population, or a subset of it, begins to characterize both individual success and how well agents are co-adapting.

As a baseline measure of coordination, we also consider wealth accumulation averaged over all *N* agents, W.

$$W=\frac{1}{N}\;\sum_{i=1}^{N}W\left(i\right)$$ (2)

Using this measure, SRR shows that co-adaptation results in significantly more agent wealth than in the non-adaptive case, i.e. when S = 1.

#### Environmental order

Another measure of successful co-adaptation is environmental order, a measure of the degree to which the environment is structured. Environmental order, *θ*, is defined as

$$\theta =1+\frac{\sum_{e=1}^{E}p\left(e\right)\text{ln}\left(p\left(e\right)\right)}{\text{ln}\left(E\right)}.$$ (3)

The numerator in the second term is the entropy of environmental states, where *p(e)* is the probability that the environment is in state *e*, averaged over some time window. When the environment is maximally disordered so that *p(e)* = 1/E for all *e*, *θ* = 0. As the *p(e)*'s move away from 1/E, the environmental order increases. If the environment is maximally ordered so that p(e*) = 1 for some e* and *p(e)* = 0 for all e≠ e*, then *θ* = 1. Order in the environment, as measured by *θ*, is often an indicator of productive mutualisms among (some of) the agents because the potential mutualisms are only realized and become stable when they collectively reduce uncertainty about future states of the environment by producing more of some of the states than others. In that way, environmental order can be thought of as a measure of the agents' success constructing a mutually understood and beneficial complex of behaviors in a malleable environment.

#### Switching rate

To track the process of co-adaption, we also consider the probability that an agent switches strategies in a given time step. Since the agents act in random order, this probability represents a uniformly sampled average over all agents of the probability that an agent switches strategies within a given time window.

### Model Behavior

**Fig 1:**
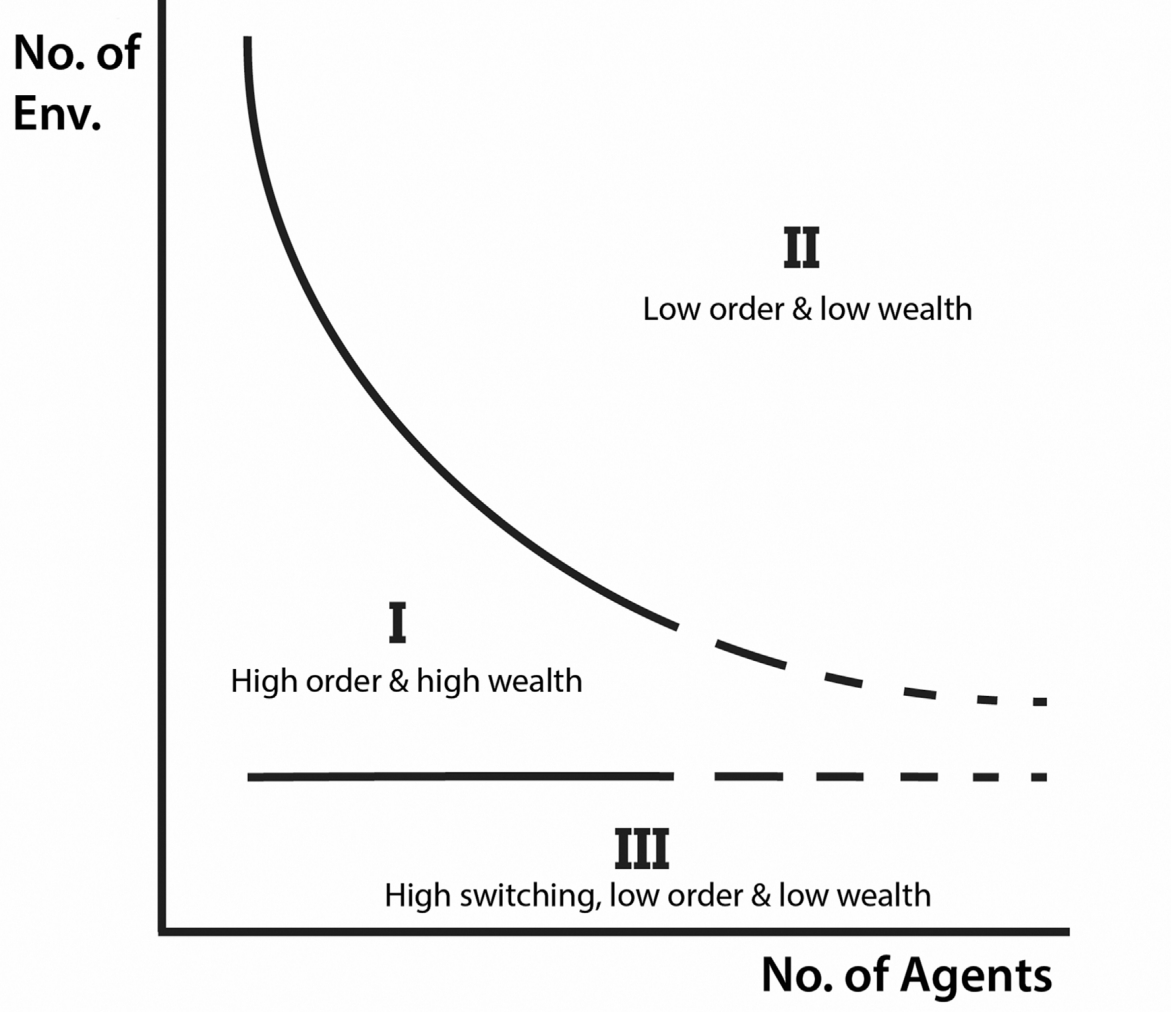
Notional Diagram. This diagram depicts three distinct regions in *NxE* parameter space of the model. Region I exhibits a moderate switching rate, high degrees of order, θ, and average agent wealths, $\bar{W}(i)$. Region II has a lower switching rate but also a lower order and average wealth than region I. Region III has a high switching rate and order and average wealth near zero.

Fig 1 is a notional diagram indicating the general behavior of the model as a function of *N* and *E* for *S* = 64. The underlying data are from 32 runs of Nx10^6^ steps. The measuring interval for all three metrics was the final *N x 10*^*4*^ time-steps of the run. In this figure, *NxE* plane is divided into three regions. In region **I**, the system generates high average agent wealth, a relatively high environmental order measure, and a moderate switching rate. In region **II**, agent wealth and environmental order is positive but noticeably lower than in region **I**. There is also a lower average switching rate. The transition between the regions is easily noticeable, but not abrupt. Finally, in region **III** there is near-zero agent wealth, a nearly unstructured environment, and a very high average switching rate. As described in SRR, in general, agents are able to coordinate their strategy choices well in region **I**, to some extent in region **II**, and not at all in region **III**. Poor coordination in region **III** is a consequence of the small number of possible reward columns in the strategies, which leads to close scores among agents’ strategies and switching among them. This is discussed in more detail in SRR.

This figure summarizes important aggregate features of the problem of co-adaptation, but those features could be produced by either homogenous or heterogeneous behaviors among the agents. In the next section we show that agents tend to segregate into two distinct groups in regions **I** and **II**. Members of one group find mutually advantageous strategies early on and cease adapting. This group molds the environment to its strategic imperatives, which results in considerably higher average wealth for those agents than for the members of the second group.

### The Dynamics of Group Formation

SRR showed it is possible for stigmergic co-adaption to lead to mutually beneficial patterns of behavior but did not study the origins and characteristics of the emergent mutualisms. In our further analysis, we found the origins of groups and differential success lie in when agents stop switching among strategies.

Visual inspection of the data showed an intriguing pattern in which several agents stop switching strategies around the same time, while the rest stop much later, if at all. We test whether the adaptive mutualisms corresponded to this separation between these two groups by first formalizing the separation.

#### In-group and out-group distinction

The in-group and out-group distinction is a natural starting point for understanding patterns of co-adaption because each additional settled (i.e. no longer switching) agent likely decreases the entropy of the system, thereby making the next state of the environment more predictable on average. One might expect the decreased environmental entropy (increased environmental order) to induce others to stop switching also, but in fact the opposite can be true, as other agents find their current strategies to be less well adapted to the new frequencies of environmental states and switch to a different strategy. This suggests a natural distinction between a set of agents who can readily accommodate (and enhance) the developing order in the environment, and those who cannot.

#### Definition of groups

In most runs, it is obvious which agents comprise the in-group and the out-group since there is a clear, unmistakable subset of agents who stop switching strategies earlier in the game. To formalize this visual intuition and accommodate occasional ambiguity, we present here a definition for robust group assignments. It places agents along the dimension of time according to when they last switched strategies and seeks to minimize the average distance in this dimension between agents within each group. For each possible assignment of *N* agents to two groups, we calculate a “distance” score as described below. The assignment with the lowest score is used to define the groups.

We denote the time agent *i* last switched as *d*_*i*_ and define *D* to be an *NxN* matrix where *D*_*i*,*j*_ is

$$D_{i,j}={\left(d_{i}-d_{j}\right)}^{2}.$$ (4)

We then define *I* to be a row vector denoting one of the 2^N^ possible assignments of agents to the in-group; the j^th^ element, x_j_, of *I* is 1 if the *j*^*th*^ agent is in the in-group and 0 otherwise. Vector *O* is the complement of vector *I* and assigns agents to the out-group such that the *j*^*th*^ element is 1 if the *j*^*th*^ agent is in the out-group and zero otherwise.

$$I=\;\{x_{1},\ldots ,x_{n}\}\;:\;x\;\in \;\{0,1\}$$ (5)

$$O=I^{c}=\{\left|I_{1}-1|,\ldots ,|I_{n}-1\right|\}$$ (6)

Let *n*_*I*_ be the number of agents in the in-group and *n*_*O*_ be the number of agents in the out-group for a particular assignment of agents to *I* and *O*. Then, the number of distinct pairs of agents in the respective groups, *S*_*I*_ and *S*_*O*_, are

$$S_{I}=\;\left(\begin{array}{c} n_{I}\\ 2 \end{array}\right)=\frac{n_{I}(n_{I}-1)}{2}$$ (7)

$$S_{O}=\;\left(\begin{array}{c} n_{O}\\ 2 \end{array}\right)=\frac{n_{O}(n_{O}-1)}{2}$$ (8)

The distance score is the sum of the average intra-group distances for a given assignment of agents to the two groups

$$\text{Score}(I)=\frac{1}{S_{I}}+\;\frac{1}{S_{o}},$$ (9)

where **1** = {1,…, 1} is used to find the sum of *N* elements in the Nx1 vector with which it is multiplied. Each of the two terms on the right hand side of Eq 9 is an average squared-distance among all pairs of agents in the same group and the best group assignment is the one with the minimum score from among all possible assignments.

In principle, this definition supports a solution in which all agents are in the same group, except in the trivial case of *N* = 2. In all of our runs, however, the definition generates both an in-group and out-group. This definition yields a clear winner, as the score of the winning assignment is an average of 6.825 standard deviations below the average of the next ten lowest scores (see S1 Appendix for a note about this calculation). This emphasizes the fact that this definition corresponds to an important feature of the co-adaptive process. We also considered three other assignment definitions to test the robustness of the group distinction. In the main, these alternative methods give similar results to the method we have chosen, but occasionally fail in cases in which the group assignments are intuitively obvious. Those alternative definitions are discussed in the S2 Appendix.

#### Wealth comparison between groups

A simple comparison of average agent wealth within each group may fail to capture significant differences in *relative* performance, as overall average wealth changes as a function of *N* and *E*. Because of this, we define a measure of context-dependent wealth. Small real differences in wealth may represent large relative differences in performance when the overall wealth is small so our metric normalizes between-group wealth differences to overall wealth accumulation for the whole group. First, we define a non-linear measure of the rate of average wealth accumulation within a group, ξ:

$$\xi =\frac{\bar{W}}{2}\left(1-\frac{\bar{W}}{2}\right)$$ (10)

where $\bar{W}$ is the average wealth of the agents in a group. Note that $\bar{W}$ and ξ are related monotonically and $-1\leq \bar{W}\leq 1$ and −3/4 ≤ *ξ* ≤ 1/4.

When ${\bar{W}}_{I}$ and ${\bar{W}}_{O}$ are the average wealth of the in-group and out-group respectively, then the difference of ξ_I_ and ξ_O_, the values of ξ for the in-group and out-group, is

$$\Delta \xi =\xi_{I}-\xi_{O}=\frac{({\bar{W}}_{I}-{\bar{W}}_{O})\left[1-\frac{{\bar{W}}_{I}+{\bar{W}}_{O}}{2}\right]}{2}.$$ (11)

The first term in the numerator is the just difference between the in-group and out-group average wealths. The second term weights that difference according to how far away the average wealth for all agents is from 1. Dividing by two normalizes Δξ so that it is bounded between -1 and 1. It is 1 if ${\bar{W}}_{I}=1$ and ${\bar{W}}_{O}=-1$, and -1 if ${\bar{W}}_{I}=-1$ and ${\bar{W}}_{O}=1$. Of course, Δξ = 0 if ${\bar{W}}_{I}={\bar{W}}_{O}$. Δξ scales scores such that a fixed difference in the group wealths, ${\bar{W}}_{I}-{\bar{W}}_{O}$, results in a smaller value if the total average group wealth, ${\bar{W}}_{I}+\;{\bar{W}}_{O}$, is larger. That is, Δξ properly captures the idea that small differences in group wealth are more significant if the total average wealth is smaller. (In the occasional case where ${\bar{W}}_{I}+\;{\bar{W}}_{O}$ is negative, differences in group wealth are accentuated, expressing the idea that not losing as much as others in a hostile environment is a significant accomplishment.)

**Fig 2:**
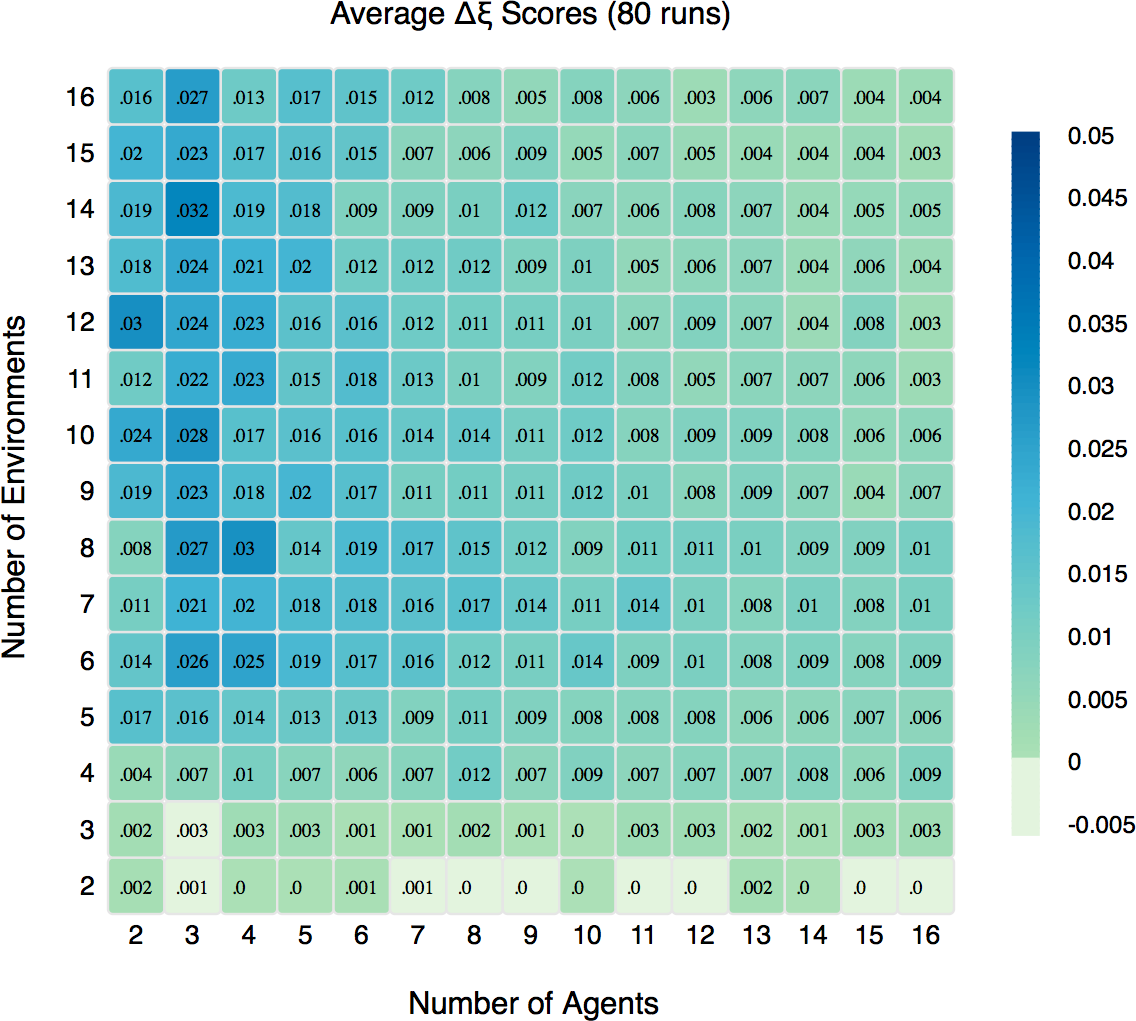
Wealth Differences: Average of Δξ for 80 runs for all combinations of *N* in [2,16] and *E* in [2,16].

**Fig 3:**
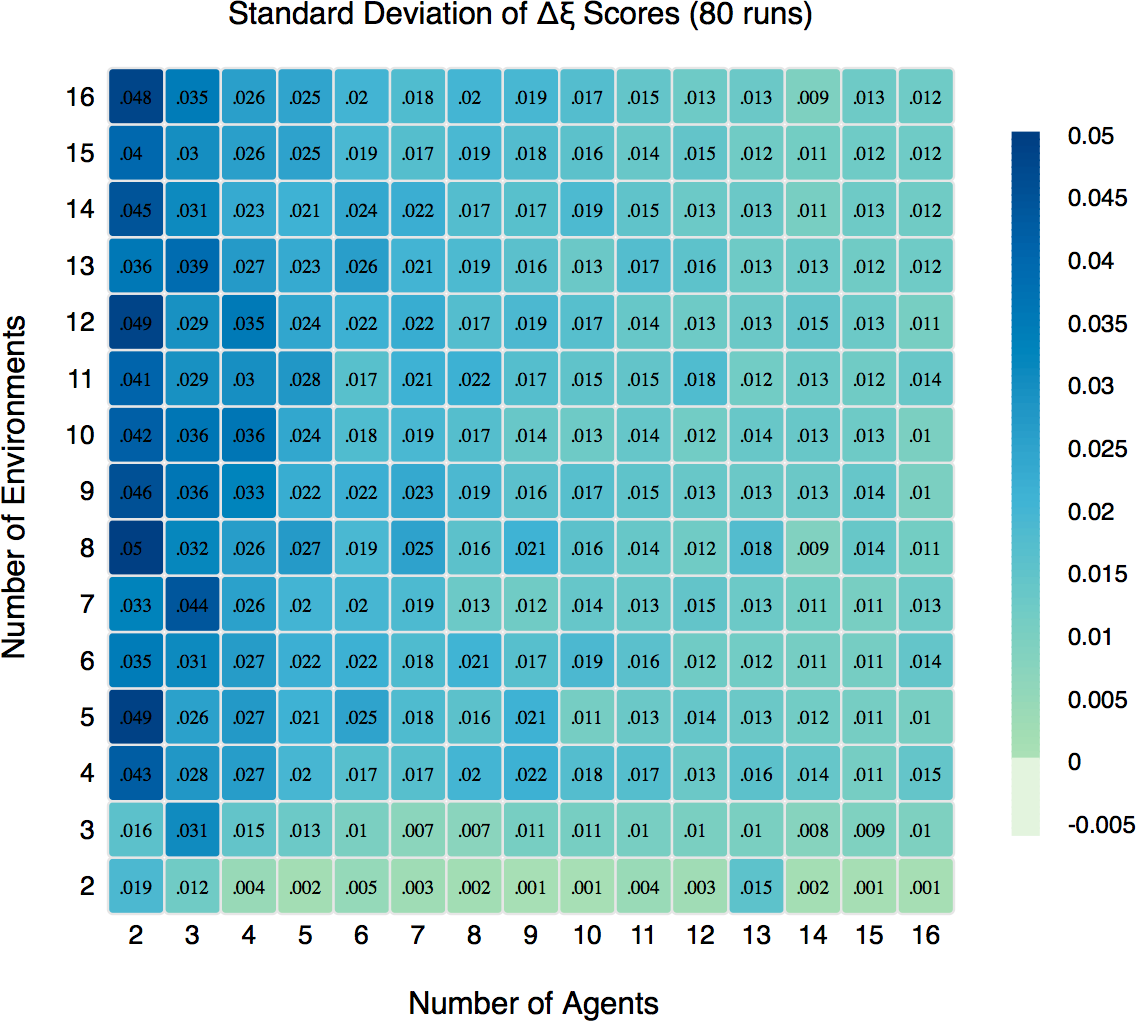
SD of Wealth Differences. For all combinations of *N* in [2,16] and *E* in [2,16], the standard deviation of the 80 Δξ scores.

To compute an average Δξ, we performed 80 runs for each combination of *N* and *E* in the range [2,16] with *S* = 16. *S*, the number of strategies, does not materially affect the results once it is large enough (S> = 10) and therefore we use S = 16 here instead of S = 64 as was the case in SRR. Each run was *N* x 10^7^ steps long, a length determined through trial and error to be well past any transient dynamics. For each run ${\bar{W}}_{I}$ and ${\bar{W}}_{O}$ were calculated using the metric for average wealth defined above (Eq 5) for the final *N* x 10^4^ steps of the run. This value captures the rate of wealth accrual after group membership for the agents has become stable. Using the winning group assignment as specified above, we calculate the Δξ for each run in our parameter space. Fig 2 shows the average Δξ of 80 runs for each *N* and *E* combination. Fig 3 shows the standard deviation.

It is clear in Fig 2 that the in-group has a consistent advantage in the rate at which its members accumulate wealth. The values of Δξ might seem modest, but the absolute numbers can be misleading. For example, if the average wealth of the out-group members per turn is .1 and the in-group average is a full 50% more at .15, the resulting Δξ is .0219. If the in-group is receiving .55 per turn and the out-group receives .50, Δξ is .0119 (but wealth accumulation at this high rate is infrequent except for very low values of *N*).

Fig 2 exhibits a pattern similar to the notional diagram in Fig 1; region **I** in the latter is where the average reward is highest and, in Fig 2, corresponds to the largest advantage for the in-group. Region **II** has lower, but still positive average reward and the in-group has a more modest advantage here. Finally, region **III** produces little, if any, rewards. This is consistent with the lack of a substantial in-group advantage for low *E* apparent in Fig 2. Interestingly, because the within-run standard deviation of wealth in the original analysis (see Fig 2A in SRR) follows roughly the same pattern as the in-group advantage, it suggests that the in-group advantage is a significant driver of the variation between agents in a run.

**Fig 4:**
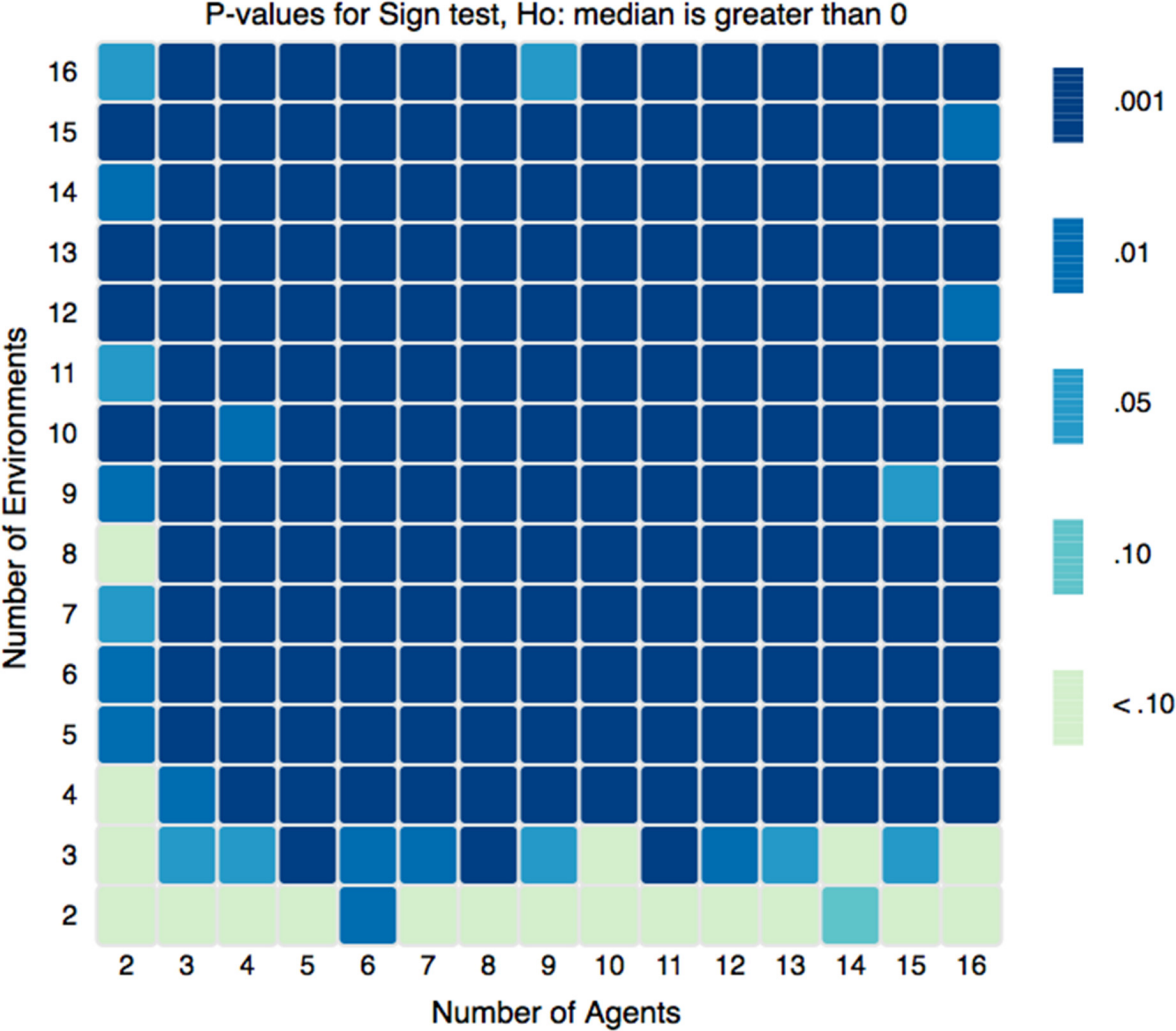
One-Sample Sign-test Results. For all combinations of *N* in [2,16] and *E* in [2,16], the significance with which we can reject the hypothesis that the median value of Δξ is equal to zero.

To test the statistical significance of the differences shown in Fig 2, we conduct a one-sample sign-test, a non-parametric test used to test the null hypothesis that the median of a distribution is equal to a given value. This test was ideal because the distribution of Δξ scores is non-normal and asymmetric. Our null hypothesis was that the sample median of Δξ, denoted as η(Δξ) was less than or equal to zero (H_O_: η(Δξ) ≤ 0). For the vast majority of combinations of *N* and *E* we can reject this null with a significance level of 0.999. Fig 3 above shows the standard deviations of the scores and Fig 4 shows the results of the one-sample sign-test. The S3 Appendix discusses these figures in more detail.

The robustness of our result that average wealth accumulation is strongly correlated with group assignment indicates that this binary group assignment algorithm captures some robust feature of the underlying dynamics of system with endogenous co-adaptation. However, one could argue that there maybe meaningful finer distinctions among the agents. For example, why not 3 or more groups, each of which is correlated with a different average wealth accumulation? For that matter, why not *N* groups, which is the same as putting each agent into his own group? (A monotonic relation between an agent’s wealth and the time it stopped switching its strategy would argue in favor of this last assignment, but we have found no evidence of this simple monotonicity.) Indeed, these may be useful starting points for analysis, but in this paper we restrict our discussion to the simple binary group assignment, a level of coarseness that has compellingly identified an important feature of the game.

Our analysis has identified one particular and important pattern present in the emergent order of the game, but there are many more avenues one could explore; do the relative sizes of the groups vary systematically? Do long-lived environmental distributions have any particular kind of characteristics? Are there systematic differences in the distribution of states produced by the two groups? Rather than turn to these other interesting questions like these, we now discuss in more detail the origins and robustness of the groups and the in-group’s earning advantages.

### Discussion and Conclusion

In this paper we have shown that agents in the stigmergy game naturally fall into two groups for a wide range of model parameters: the first, the in-group, consists of agents who settle on a strategy relatively early in the game; the second, the out-group, consists of all other agents who continue to switch their strategies well into the course of the run. Importantly, the functional relevance of these groups is seen in the fact that members of the in-group consistently earn higher rewards than do the members of the out-group and this is the chief feature that distinguishes the groups. This result has a number of implications and raises several interesting questions.

First, it is striking that those agents who do better are the ones who have ceased to change their strategies. This is a consequence of the endogenous way the environment is structured and the stigmergic signaling among the agents. When an agent consistently plays one strategy, it creates a predictable environmental signal and will therefore have stronger influence on the structure of the environment than otherwise. Thus, from the point of view of emergent coordination, an agent with consistent responses to environmental signals will be more easily “read” by other agents and it will thus be easier to develop cooperative behaviors that benefit these behaviorally stable agents. This result is not necessarily what one would have expected. After all, even late in the game, continuing to adapt should allow agents to better respond to the distribution of environmental states. But this intuition that the more adaptive agents should be the better performers might only make sense in the context of an environment that is not primarily generated endogenously. If changes to the environment are primarily produced exogenously, then, indeed, the most agile agents generally should be the ones best able to adapt to the changing environment. That intuition, however, is not borne out when agents are able to form tacit groups whose behaviors materially affect the structure of the environment itself.

The result that members of the in-group do better than members of the out-group also raises the question of whether there is something intrinsic in the strategies of in-group members that results in better performance, or if that better performance is arbitrary or otherwise depends on the complex dynamics of endogenous co-adaptation. We address this question in S4 and S5 Appendices by analyzing the respective roles of agents’ strategies and the order in which agents act in producing the outcomes we observe. We find that the outcomes are not exclusively a consequence of the agents’ strategies—what we call an “endowment effect”—because the game is path dependent in so far as changing the order in which agents act leads to different outcomes. To show this we perform several runs with the same sets of strategies for the individual agents but different sequences of agent moves. We find different outcomes among runs in terms of average wealth, environmental entropy, agent group assignments, and wealth difference between the in-group and the out-group of the run, although within each run the qualitative structure of game remains; namely, the in-group generally does better than the out-group regardless of the composition of the group. (See the S4 Appendix for the details of the analysis.) This evidence does not preclude features of the strategies playing a role in outcomes, but it does show that the dynamics of interaction are *path dependent* and play a central role in the final outcome of the game. Thus, the above finding that those agents who settle on a strategy earlier receive benefits at a higher rate than others is primarily a claim about the dynamics of coordination and niche construction and not about the nature of the agents themselves. That said, there is a correlation between the nature of an agent`s strategies and the likelihood that that agent will be a member of the in-group. Some agents are much more likely to belong to one or the other group than one would expect if the group assignments were random. This finding is discussed in the S5 Appendix. Taken together, these two results highlight the fascinatingly complex nature of the dynamics of the stigmergy game.

There may also be implications of our work for the ecological stability of a system. Systems with endogenous co-adaptation may have very different responses to exogenous shocks than those in which the structure of the environment is largely immune to the actions of the agents. In the case in which agents’ actions do not materially affect the environment, the response to an exogenous change in the environment will not involve any feedback to the environment itself. However, in situations in which agents’ actions materially affect the environment (and to which the stigmergy game is applicable) there is an additional dynamic that may try to”heal” the environment. One might suppose that this additional “healing” dynamic would help agents to respond better to an exogenous shock to the environment, therefore rendering the overall system behavior more robust. But that is far from clear. Understanding robustness and ecological stability in light of such endogeneity is an important goal given how many adaptive systems include it.

Finally, while there are relatively few systems for which stigmergy is the exclusive mechanism of communication, it is clearly an important mode of communication among co-adapting agents in a large variety of systems. This has implications for much of the research on complex adaptive systems, and for work on inter-agent communication, particularly in the context of networks. While recent work on networks has provided many important insights, for many systems, an exclusively network-based analysis of inter-agent communication and interaction may not be the most appropriate and convenient one, since it draws attention away from the more diffuse and less specific information that enables stigmergic co-adaptation.

In the context of a game in which the environment is endogenously produced and there is only stigmergic communication among the co-adaptive agents, we have shown that there is an intrinsic dynamic that generates two groups, and that the in-group does better at amassing resources than does the out-group. This differentiation is not directly attributable to differences between agents' strategies and is largely the result of fortuitous happenstance (although we did find evidence that some agents do tend to end up in one of the groups more often than random assignment would predict). These intriguing insights only underline the need for a fuller understanding of how endogenous co-adaptation proceeds. Insofar as endogenous molding of the environment and stigmergic feedback is common in complex adaptive systems, this is an important dynamic that requires more extensive study.

## Supporting Information

S1 Appendix Group Definition Robustness. (DOCX)

S2 Appendix Alternative Group Definitions. (DOCX)

S3 Appendix Standard Deviations and Sign Test. (DOCX)

S4 Appendix Path Dependence. (DOCX)

S5 Appendix Endowment Effect. (DOCX)

## References

[1] Herrnstein R, Murray C. The Bell Curve: Intelligence and Class Structure in American Life New York: Free Press; 1994.

[2] Pager D, Shepherd H. The Sociology of Discrimination: Racial Discrimination in Employment, Housing, Credit, and Consumer Markets. Annu. Rev. Sociol. 2008;34: 181–209. 20689680 10.1146/annurev.soc.33.040406.131740 PMC2915460

[3] Katznelson I. When Affirmative Action was White New York: W.W. Norton & Company; 2005.

[4] Heckmann JJ. The Economics, Technology, and Neuroscience of Human Capability Formation. Proc Natl Acad Sci USA. 2007;104(33): 13250–13255. 17686985 10.1073/pnas.0701362104 PMC1948899

[5] Keister L, Moller S. Wealth Inequality in the United States. Annu. Rev. Sociol. 2000;26: 63–81.

[6] Marx K. Capital, Volume 1 New York: W.W. Norton; 1978.

[7] DiPrete T, Eirich GM. Cumulative Advantage as a Mechanism for Inequality: A Review of Theoretical and Empirical Developments. Annu. Rev. Sociol. 2006;32: 271–297.

[8] Piketty T. Theories of Persistent Inequality and Intergenerational Mobility In: Atkinson A, Bourguignon F, editors. Handbook of Income Distribution, Volume 1 Amsterdam: North-Holland; 2000 pp. 430–476.

[9] Savit R, Riolo M, Riolo R. Co-Adaptation and the Emergence of Structure. PLoS One. 2013;8(9): e71828 10.1371/journal.pone.0071828 24039722 PMC3769280

[10] Odling-Smee J, Laland K, Feldman M. Niche Construction: The Neglected Process in Evolution Princeton: Princeton University Press; 2003.

[11] Pigliucci M, Müller GB. Evolution: The Extended Synthesis. Cambridge: MIT Press; 2010.

[12] Laland K, Uller T, Feldman M, Sterelny K, Müller GB, Moczek A, et al Does Evolutionary Theory Need a Rethink? Nature. 2014;514: 161–164. 10.1038/514161a 25297418

[13] Anderson JM. Soil Organisms as Engineers: Microsite Modulation of Macroscale Process In: Jones CG, Lawton JH, editors. Linking Species and Ecosystems. New York: Chapman and Hall; 1995 pp. 94–106.

[14] Henschel JR. Tool Use By Spiders: Stone Selection and Placement By Corolla Spiders Ariadana (Segestriide) of the Namib Desert. Ethology. 1995;101(3): 187–199.

[15] Parziale L, Britt D, Davis C, Forrester J, Liu W, Matthews C, et al TCP/IP Tutorial and Technical Overview IBM Redbooks: Internal Technical and Support Organization; 2006.

[16] Systems Cisco. Routing Basics: Link-State Versus Distance Vector. Internetworking Technology Handbook 2012 Available: http://docwiki.cisco.com/wiki/Routing_Basics

[17] Altman E, Avrachenkov K, Bonneau N, Debbah M, El-Azouzi R, Menasche D. Constrained Stochastic Games in Wireless Networks. Global Telecommunications Conference. 2007; 315–320.

[18] Smith RD. The Dynamic of Internet Traffic: Self-Similarity, Self-Organization, and Complex Phenomena. Adv Complex Syst. 2011;14(6): 905–949.

[19] Peirce CS. Peirce on Signs: Writings on Semiotic by Charles Sanders Peirce Chapel Hill: University of North Carolina Press; 1991.

[20] Goldman M, Schurman R. Closing the “Great Divide”: New Social Theory on Society and Nature. Annu Rev Sociol. 2000;26(1): 563–584.

[21] Keane W. Semiotics and the Social Analysis of Material Things. Lang Commun. 2003;23(3): 409–425.

[22] Durkheim E. The Elementary Forms of Religious Life Oxford: Oxford University Press; 2001.

[23] Barthes R. Elements of Semiology New York: Hill and Wang; 1977.

[24] Blumer H. Symbolic Interactionism: Perspective and Method Berkeley: University of California Press; 1986.

[25] White H. Where Do Markets Come From? Amer Journ of Socio. 1981;87(3): 517 547.

[26] Carroll G. Concentration and Specialization: Dynamics of Niche Width in Populations of Organizations. Amer Journ Socio. 1985;90(6): 1262–1283.

[27] Podolny J. A Status-based Model of Market Competition. Amer Journ Socio. 1993;98(4): 829–872.

[28] Padgett J, Powell W. The Emergence of Organizations and Markets Princeton: Princeton University Press; 2012.

[29] Centola D, Baronchelli A. The Spontaneous Emergence of Conventions: An Experimental Study of Cultural Evolution. Proc Natl Acad Sci USA 2015;112(7): 1989–1994. 10.1073/pnas.1418838112 25646462 PMC4343158

[30] Worden L, Levin S. Evolutionary escape from the prisoner's dilemma. J Theor Biol. 2007;245(3): 411–422. 17169377 10.1016/j.jtbi.2006.10.011

[31] Roughgarden J. The Genial Gene: Deconstructing Darwinian Selfishness Berkeley: University of California Press 2009.

[32] Akçay E, Roughgarden J. Evolution of payoff matrices: Providing incentives to cooperate. Proc R Soc Lond B Biol Sci. 2011; 278(1715): 2198–2206. 10.1098/rspb.2010.2105 PMC3107625 21147797

[33] Grasse PP. La Reconstruction du Nid et Les Coordinations Interindividuelles chez ‘Bellicositermes Natalensis et Cubitermes Sp’. La Theorie de la Stigmergie: Essai D’interpretation du Comportement des Termites Constructeurs. Insectes Soc 1959;6(1): 41–80. French.

[34] Theraulaz G, Bonabeau E. A Brief History of Stigmergy. Artif Life. 1999;5(2): 97–116. 10633572 10.1162/106454699568700

[35] Holland O, Melhuish C. Stigmergy, Self-Organization, and Sorting in Collective Robotics. Artif Life. 1999;5(2): 173–202. 10633575 10.1162/106454699568737

