# Correction: The Emergence of Groups and Inequality Through Co-Adaptation

**DOI:** 10.1371/journal.pone.0165352

**Published:** 2016-10-18

**Authors:** Jon Atwell, Robert Savit

The DOI and URL provided in the Data Availability Statement for this paper are incorrect. The correct statement is: Simulation code and the data used for analysis are available from the Deep Blue Data database through the University of Michigan Library (DOI: 10.7302/Z2Q81B0K, URL: https://deepblue.lib.umich.edu/data/concern/generic_works/5x21tf430).
