# Supplementary material for: The Emergence of Groups and Inequality through Co-Adaptation
Source: PLoS One. 2016 Jun 30;11(6):e0158144. doi: 10.1371/journal.pone.0158144 (PMC4928893; doi:10.1371/journal.pone.0158144)
Supplement: S1 Appendix — (DOCX) [file pone.0158144.s001.docx]

**S1 Appendix**

The definition for group assignments we use exhibits a significant difference between the best (minimum) score and the average of the ten next-best scores, the former being an average of 6.825 standard deviations below the latter. All cases with *N*=2 are ignored in this calculation because only two assignments are possible. Also, there are only eight possible group assignments for *N*=3 so there are only seven next-best scores used in the calculation.
