# Supplementary material for: The Emergence of Groups and Inequality through Co-Adaptation
Source: PLoS One. 2016 Jun 30;11(6):e0158144. doi: 10.1371/journal.pone.0158144 (PMC4928893; doi:10.1371/journal.pone.0158144)
Supplement: S2 Appendix — (DOCX) [file pone.0158144.s002.docx]

**S2 Appendix: Alternative Group Definitions**

Equations 7 through 12 in the Main Text (p. 16-17) define the groups we used in our analysis. We chose this definition after comparing it to three other obvious choices. The first alternative used the absolute value of the distances between agents in the definition of *D_i,j_* (Equation 7) , which deemphasizes larger differences. Another takes the square root of each of the two terms on the right-hand side of Equation 12 to reduce the penalty for larger within-group distances present in our definition. The final alternative finds the “center of mass” for the whole collection of agents along the dimension of time last switched (d*_i_*) and partitions the agents into groups at that point. Each of the alternatives mostly agrees with definition we have chosen, but also sometimes fails to correctly identify the in-group and the out-group assignment when it is visually apparent.
