# Supplementary material for: The Emergence of Groups and Inequality through Co-Adaptation
Source: PLoS One. 2016 Jun 30;11(6):e0158144. doi: 10.1371/journal.pone.0158144 (PMC4928893; doi:10.1371/journal.pone.0158144)
Supplement: S3 Appendix — (DOCX) [file pone.0158144.s003.docx]

***S3 Appendix: Standard Deviation of Δξ scores and Statistical Test***

Figure 3 reports the standard deviations of the $\Delta\xi$ scores used to create Figure 2, the average $\Delta\xi$scores. For each combination of *N* and *E* there were 80 unique runs.

We use this same collection of runs to test the hypothesis that the in-group wealth advantage is greater than zero *for each combination of N and E, a total of 225 unique tests.* We use a one-sample sign-test, a non-parametric test with a null hypothesis that the population median is different than a specified value. The more standard one-sample t-test is not appropriate because visual inspection of the distributions revealed them to be non-normal and asymmetric. Our null hypothesis was that the population median is equal to zero, while the alternative hypothesis was that it is greater than zero. The significance level with which we can reject the null is reported in Figure 4.

As one can see, for most combinations of *N* and *E* we can reject the null with a significance level of .001. This strongly supports the conclusion that the Δξ are positive and that the in-group has an earnings advantage. There are, however, two regions in which the significance level is lower. For N=2, there is always one agent in each group, but they often find an equilibrium with maximum and identical rewards thereby ensuring that Δξ= 0. For *E*=2 and *E*=3 there are a small number of possible strategies and therefore agents are likely to have very similar or even identical strategies. This leads agents to continually alternate among those similar strategies and doing so prevents the whole collection of agents from effectively coordinating. These regions notwithstanding, a positive average Δξ is common and statistically robust.
