# Supplementary material for: The Emergence of Groups and Inequality through Co-Adaptation
Source: PLoS One. 2016 Jun 30;11(6):e0158144. doi: 10.1371/journal.pone.0158144 (PMC4928893; doi:10.1371/journal.pone.0158144)
Supplement: S4 Appendix — (DOCX) [file pone.0158144.s004.docx]

**S4 Appendix: Path Dependence.**

There are only two sources of randomness in the stigmergy model: agents` strategies, which are based on randomly generated lists, and the order of agents` turns, which is fully random. To better understand the relative importance of the latter in generating the observed variation between runs for the same *N*, *E* and *S*, we first look for path dependence within the model. A system exhibits path dependence when outcomes depend on the order of events in a dynamic process (not to be confused with depending on the occurrence of specific events [Page, 2006]). We hypothesize path dependency can explain some of the variation in outcomes we observe with the stigmergy game. More specifically, we hypothesize that when the same agents move in a different random order, features of the emergent order will be different. To test this, we run the model multiple times for agents with the same sets of strategies, but with the agents acting in a different order. This allows us to study the effect of order in isolation because if the emergent features vary across runs with the same agents, it must be the case that the order of actions, at least in part, determines the outcomes of the model.

We do not attempt to quantify the exact degree of path dependency but instead simply establish its presence. Our procedure for doing is as follows: For a variety of combinations of *N* and *E* (with *S*=16), we created 12 instances of the model (now referred to as “cases”) with *different* agents. We then ran each case with 30 different random-number-generator seeds for determining the order in which agents act and recorded the average wealth [*W(i)*], environmental order (θ), group wealth differences (Δξ), and the group assignment vectors for each run in order to construct metrics for comparing the runs within a case. Our metrics are reported in Table A; we now define them.

|  | Measures | N=4 | N=8 | N=12 | N=16 |
| --- | --- | --- | --- | --- | --- |
| E=4 | Fract - Wealth - μ, σ  Fract - Order - μ, σ  Δξ - GM(μ _i_), SD(μ _i_)  Hamming - GM(μ _i_), max | 0.546, 0.289  0.112, 0.107  0.021, 0.015  1.583, 4.0 | 0.439, 0.168  0.012, 0.009  0.016, 0.006  2.801, 7.5 | 0.533, 0.284  0.011, 0.007  0.017, 0.006  3.934, 11.25 | 0.479, 0.202  0.008, 0.003  0.016, 0.005  5.097, 13.417 |
| E=8 | Fract - Wealth - μ, σ  Fract - Order - μ, σ  Δξ - GM(μ _i_), SD(μ _i_)  Hamming - GM(μ _i_), max | 0.514, 0.229  0.092, 0.128  0.03, 0.011  1.545, 3.833 | 0.568, 0.141  0.006, 0.002  0.02, 0.008  3.175, 6.917 | 0.507, 0.205  0.005, 0.002  0.014, 0.004  4.339, 10.5 | 0.466, 0.127  0.003, 0.001  0.012, 0.003  5.851, 13.417 |
| E=12 | Fract - Wealth - μ, σ  Fract - Order - μ, σ  Δξ - GM(μ _i_), SD(μ _i_)  Hamming - GM(μ _i_), max | 0.349, 0.16  0.017, 0.013  0.025, 0.011  1.303, 3.75 | 0.32, 0.084  0.005, 0.001  0.019, 0.007  3.237, 7.167 | 0.346, 0.118  0.003, 0.001  0.014, 0.002  5.148, 11.417 | 0.359, 0.061  0.003, 0.001  0.014, 0.001  6.751, 15.0 |
| E=16 | Fract - Wealth - μ, σ  Fract - Order - μ, σ  Δξ - GM(μ _i_), SD(μ _i_)  Hamming - GM(μ _i_), max | 0.398, 0.182  0.01, 0.003  0.03, 0.008  1.556, 4.0 | 0.414, 0.112  0.005, 0.001  0.02, 0.005  3.526, 7.833 | 0.415, 0.093  0.003, 0.001  0.014, 0.003  5.269, 11.25 | 0.386, 0.07  0.002, 0.001  0.014, 0.002  7.236, 14.833 |

Table A: Measures of Path Dependency

Because average wealth and environmental order varies in *N* and *E* and because there might be unaccounted-for differences between sets of strategies in the 12 different cases, we construct normalized measures. For both measures we find the absolute value of the differences for all 435 pairs of runs within a single case and then divide those differences by the average value for that case to get a *fractional difference* between pairs of runs for the case. We then find the average fractional difference of the value across all pairs of runs in the case. The average fractional differences within a case for *W(i)* and θ are shown in Equations S1 and S2, respectively, for pairs of runs indexed by *k* and *l*.

${W(i)}_{\text{frac}}= \frac{1}{435} \sum_{1}^{435} \frac{| {W\left( i \right)}_{k}- {W\left( i \right)}_{l} |}{\frac{1}{30} \sum_{j=1}^{30} {W(i)}_{j}}$ (S1)

$\theta_{\text{frac}}= \frac{1}{435} \sum_{1}^{435} \frac{| \theta_{k}- \theta_{l} |}{\frac{1}{30} \sum_{j=1}^{30} \theta_{j}}$ (S2)

We report the mean and standard deviation of these two metrics for the 12 cases for each combination of *N* and *E* in Table A.

Our definition of Δξ is already normalized according to overall wealth within a run, so we report the grand mean (the mean of 30-run means for the 12 cases) and the standard deviation of the case means in Table A. Finally, we recorded the group assignments for each run and calculated the Hamming distance between assignments for all pairs of runs in a case. This is equivalent to the number of switches in assignments between pairs of runs; it allows us to quantify how much the makeup of the in-group and out-group is changing between runs. We report the grand mean of this quantity. For each run, we also identified the maximum distance from among its 29 pairings to highlight the full extent of the variation. We find the average maximum distance for each case and then report the grand mean of the 12 cases.

Table A shows significant differences in run-wealth relative to overall wealth (the average fractional differences, the first row in each cell of the table). The average values range between .32 and .568, meaning that the difference in overall wealth between any pair of runs in a given case is high relative to the average overall wealth for that case. For example, if the average fractional difference is .50 and the average wealth for the case is .40, the typical difference in wealth between a pair of runs is .20. These significant differences appear to be quite common within cases and show that changing the order of agent turns can drastically alter this important emergent feature of the game. Additionally, the mean Hamming distances between assignments are healthily positive. The fourth row in each cell shows an average of between a quarter and half of agents changing group assignments when the order of agent moves is changed. The maximum differences also come close to the total number of agents, meaning that some pairs of runs within a case have nearly opposite assignments. Thus, the order of agent moves is crucial in determining which agents are in the in-group. Taken together with the variation in average wealths, this convincingly shows the stigmergy game has an abundance of path dependency.

Possible counterevidence to this claim is the fact that the fractional differences in our measure of order, $\theta$, tend to be smaller than the fractional differences in wealth. This is not that surprising, however, because we use a standard measure of entropy in the definition and therefore the measure can yield the same score for different arrangements of the same environmental frequencies. This measure cannot quantify the order of environmental states, but merely orderedness; that we see positive fractional differences in $\theta$ at all is more evidence of path dependency.

Finally, the variation in the case means of Δξ is generally small. The grand means are also similar to the corresponding values in Fig 2. This is important evidence that the in-group advantage is a truly emergent feature of the game; regardless of the overall wealth, order, and composition of the groups, the members of the in-group do better on average than the members of the out-group. So while random events early in the game determine some important facts about the outcome, the fact that the in-group has an advantage does not change.
