# Supplementary material for: The Emergence of Groups and Inequality through Co-Adaptation
Source: PLoS One. 2016 Jun 30;11(6):e0158144. doi: 10.1371/journal.pone.0158144 (PMC4928893; doi:10.1371/journal.pone.0158144)
Supplement: S5 Appendix — (DOCX) [file pone.0158144.s005.docx]

**S5 Appendix: Endowment Effect.**

The evidence of path dependence presented in the S4 Appendix is compelling evidence that the order in which agents act is an important factor in determining outcomes. This does not, however, preclude the possibility that outcomes depend in part on the strategies agents have, either as a collective or as individuals, a feature we refer to as an “endowment effect.“ Table B reports several metrics, defined below, that suggest outcomes do in fact depend in part on the randomized strategies agents have. The first three metrics we present attempt to capture whether the collection of all strategies in the group can lead to different average wealth (*W(i)*), environmental order (θ), and group wealth differences (Δξ). Our strategy is to compare the 30-run distribution of outcomes for each of the 12 cases for a given *N* and *E*. We have already established there is variation in outcomes for the same set of strategies, but not that the variation looks the same for different sets of strategies. If the distributions are not the same, it suggests that the general dynamics of the game are not independent of the randomized set of strategies agents have, and an endowment effect must be present.

|  | Measures | N=4 | N=8 | N=12 | N=16 |
| --- | --- | --- | --- | --- | --- |
| E=4 | *W*: Pr(reject H_o_)  Θ: Pr(reject H_o_)  Δξ: Pr(reject H_o_)  5% tails, in-group probability (μ,σ) | 0.894  0.939  0.818  0.625, 0.26 | 0.788  0.833  0.667  0.521, 0.233 | 0.773  0.758  0.515  0.535, 0.146 | 0.773  0.727  0.576  0.49, 0.194 |
| E=8 | *W*: Pr(reject H_o_)  Θ: Pr(reject H_o_)  Δξ: Pr(reject H_o_)  5% tails, in-group probability (μ,σ) | 0.909  0.894  0.53  0.771, 0.216 | 0.652  0.697  0.394  0.656, 0.235 | 0.788  0.803  0.439  0.625, 0.203 | 0.712  0.697  0.485  0.5, 0.125 |
| E=12 | *W*: Pr(reject H_o_)  Θ: Pr(reject H_o_)  Δξ: Pr(reject H_o_)  5% tails, in-group probability (μ,σ)) | 0.864  0.894  0.848  0.729, 0.216 | 0.545  0.712  0.591  0.635, 0.157 | 0.697  0.545  0.409  0.465, 0.181 | 0.333  0.47  0.167  0.448, 0.127 |
| E=16 | *W*: Pr(reject H_o_)  Θ: Pr(reject H_o_)  Δξ: Pr(reject H_o_)  5% tails, in-group probability (μ,σ) | 0.697  0.652  0.697  0.75, 0.25 | 0.47  0.394  0.212  0.5, 0.135 | 0.53  0.5  0.47  0.382, 0.172 | 0.182  0.212  0.197  0.37, 0.164 |

**Table B: Endowment Effect**

To establish such an effect, we compare the 30-run distribution of outcomes for each of the 12 cases. We calculate the Kolmogorov-Smirnov statistic for pairs of distributions and record the *frequency* with which we can reject the null hypothesis that they came from the same distribution with a significance level of .95. We report this frequency for *W(i)*, θ and Δξ. The null is rejected with high frequency, and this shows that, while there is a range of outcomes for each case (as evidenced in Table A of the S4 Appendix), the distribution of outcomes is different for different cases and the sets of strategies likely have important differences.

The final metric considers how often each agent is assigned to the in-group in the 30 runs in each case. If there were no endowment effect, then we would expect that, in a given case, the probability that an agent is in the in-group will be consistent with a random (IID) assignment of agents to the in-group accounting for the overall size of the in-group for that combination of *N* and *E*. We compute a base distribution of the probability to be in the in-group among the 30 runs in a given case and compare the actual assignments of each agent to that base distribution.

The base distribution is calculated as follows: For each of the 30 runs of a given case, we note the size of the in-group, which determines the probability *p* for an agent to be in the in-group for that run. We then numerically compute the probability of an agent being assigned to the in-group *n* times assuming random assignments with probability *p*. For example, if *N*=8 and there happen to be three in-group agents for a run, one could assume there are always three agents in the in-group. However, because the stochastic elements of the game clearly matter, we assume each agent becomes an in-group member with probability .375. We assign eight agents to the in-group with a probability of .375 one-hundred times to create a distribution of in-group sizes for that run. Repeating this for each of the 30 runs in a given case, we generate an overall probability distribution of being randomly assigned to the in-group for that case. (This procedure accounts for the fact that the in-group sizes may be different for different runs in a given case.) We then note the number of times an agent would need to be assigned to the in-group in the 30 runs in order to be above the 95^th^ percentile or below the 5^th^ percentile of this distribution. We find the number of agents who were above the 95^th^ percentile or below the 5^th^ percentile based on their actual assignments. Finally, for the combinations of *N* and *E*, we report the grand mean (the mean of the twelve different 30-run case means) and standard deviation of the fraction of agents who appeared in one group more frequently than expected according to our null model.

These quantities are reported in the last subrows in Table B. They make clear that the real frequency of membership in the respective groups is greater than the probability of being randomly assigned to them, often significantly so. More specifically, the distributions of the actual assignments have much heavier tails than those of the random distribution. This strongly suggests the existence of an individual-level endowment effect for at least some agents. Taken together, the results shown in Table B indicate that, considered as a collective, the particular assignments of strategies to the agents do affect the general outcome of the game (not surprisingly), and that at least some agents possess strategies, that, relative to the strategies of the other agents, tend to make them members of the in-group even as path dependency pushes the collective outcome in different directions.

The existence of the endowment effect raises a question about the features of certain strategies, or a set of them: what gives certain agents a durable advantage? All strategies have an expected reward of zero, so the most obvious source of advantage is already controlled for. Our preliminary inquiries into this question has shown the answer to be quite complex and therefore beyond the scope of this paper.

Reference:

Page SE. Path Dependence. *Quart J Polit Sci.* 2006;1(1): 87-115.
